# Chemical Constituents of Essential Oils Used in Olfactory Training: Focus on COVID-19 Induced Olfactory Dysfunction

**DOI:** 10.3389/fphar.2022.835886

**Published:** 2022-06-02

**Authors:** Sachiko Koyama, Thomas Heinbockel

**Affiliations:** ^1^ Department of Chemistry, College of Arts and Sciences, Indiana University, Bloomington, IN, United States; ^2^ Department of Anatomy, College of Medicine, Howard University, Washington, DC, United States

**Keywords:** COVID-19 induced olfactory dysfunction, olfactory training, essential oils, chemical constituents, anti-inflammation, binding affinity with SARS-CoV-2

## Abstract

The recent increase in the number of patients with post-viral olfactory dysfunction (PVOD) following the outbreak of COVID-19 has raised the general interest in and concern about olfactory dysfunction. At present, no clear method of treatment for PVOD has been established. Currently the most well-known method to improve the symptoms of olfactory dysfunction is “olfactory training” using essential oils. The essential oils used in olfactory training typically include rose, lemon, clove, and eucalyptus, which were selected based on the odor prism hypothesis proposed by Hans Henning in 1916. He classified odors based on six primary categories or dimensions and suggested that any olfactory stimulus fits into his smell prism, a three-dimensional space. The term “olfactory training” has been used based on the concept of training olfactory sensory neurons to relearn and distinguish olfactory stimuli. However, other mechanisms might contribute to how olfactory training can improve the recovery of the olfactory sense. Possibly, the essential oils contain chemical constituents with bioactive properties that facilitate the recovery of the olfactory sense by suppressing inflammation and enhancing regeneration. In this review, we summarize the chemical constituents of the essential oils of rose, lemon, clove, and eucalyptus and raise the possibility that the chemical constituents with bioactive properties are involved in improving the symptoms of olfactory dysfunction. We also propose that other essential oils that contain chemical constituents with anti-inflammatory effects and have binding affinity with SARS-CoV-2 can be new candidates to test their efficiencies in facilitating the recovery.

## Introduction

Olfactory training to facilitate the recovery from olfactory dysfunction has received broader attention following the outbreak of COVID-19. Many COVID-19 patients have lost their sense of smell (anosmia) or experienced a decrease in their olfactory sense (hyposmia). Olfactory training has been used to facilitate the recovery of the olfactory sense in patients with olfactory dysfunction since the beginning of the 21st century after the publication of a landmark paper by Hummel et al. (2009). The effects of olfactory training on improving the sense are now much better known than in the pre-pandemic years.

As the name implies, the concept of “olfactory training” is to “train” the olfactory sensory neurons (OSNs) to, for example, “relearn what a rose smells like” (https://www.bcm.edu/news/relearning-to-smell-after-covid-19). Olfactory training uses essential oils of rose, lemon, clove, and eucalyptus based on the study by Hummel et al. (2009), which was grounded in the “odor prism” hypothesis proposed by Henning in 1916 (Henning 1916). The odor prism hypothesis is classifying the odors into flowery, foul, fruity, aromatic, burnt, and resinous. For this reason, rose (representing flowery), lemon (fruity), aromatic (cloves), and resinous (eucalyptus) became the essential oils to be used in olfactory training.

Multiple possibilities might be involved in the mechanisms that underlie olfactory “training” to facilitate the recovery of our olfactory sense. One such possibility is the influence of the chemical constituents with bioactive properties of these essential oils. In this review, we summarize the major chemical constituents with bioactive properties in these essential oils that may contribute to enhancing regeneration and facilitating the recovery of olfactory sensory function.

## Olfactory Neuroscience and Anosmia/Hyposmia

The study of our olfactory sense has seen an increase during recent years with the realization that the olfactory system can be a marker of neurodegeneration in aging, neurological and neuropsychiatric disorders (reviewed in Bhatia-Dey and Heinbockel 2021). Our olfactory sense is relevant for day-to-day behavior as well as for our quality of life (Mann, 2002; Croy et al., 2014; McGann, 2017). It has been shown that declining olfactory acuity and olfactory deficits emerge either as very first symptoms or as prodromal symptoms of progressive neurodegeneration. Most neurodegenerative, neuropsychiatric and communication disorders have olfactory dysfunction associated with them. Specifically, a decline in olfactory function is a preliminary indicator of classical neurodegenerative disorders such as Alzheimer’s Disease (AD) and Parkinson’s Disease (PD). Impaired olfaction can serve as prodromal symptom neuropathology associated with both conditions (Rey et al., 2018; Bhatini et al., 2019; Li et al., 2019).

The principal function of the olfactory system is the detection and processing of olfactory signals. This plays a critical role in physiological as well as emotional homeostasis as it relates to reproductive and neuroendocrine regulation. In humans, the olfactory pathway starts in nasal olfactory structures that detect odorants in the air (reviewed in Heinbockel and Straiker, 2021). In the nasal cavity, the olfactory epithelium houses a large population of olfactory sensory neurons (OSNs) with odorant receptor proteins in the membranes of their dendritic cilia. The olfactory signal, in the form of odorant molecules, is transduced into an electrical one, and nerve impulses are sent to the first central relay for olfactory processing, namely, the olfactory bulb (Shipley and Ennis 1996; Ennis et al., 2007; Heinbockel & Heyward 2009).

OSNs are ciliated bipolar neurons with odorant receptor proteins in the membranes of the cilia in order to bind and detect odorant molecules (Bushdid et al., 2014). The axons of OSNs form the olfactory nerve (cranial nerve I) and project through the cribriform plate of the ethmoid bone to the olfactory nerve layer of the ipsilateral olfactory bulb. The air that we breathe in is filled with odorant molecules that activate olfactory receptor proteins. Each OSN expresses one olfactory receptor gene which codes for an odorant receptor protein (Hanchate et al., 2015; Tan et al., 2015). The type of odorant receptor protein in an OSN determines its ability to detect specific odorants. The genes that encode odorant receptor proteins form a large gene family of G-protein coupled receptors (Buck and Axel, 1991; Young et al., 2002; Axel 2005; Buck 2005). In mice, this odorant receptor multigene family includes more than 1,400 genes with about 300 pseudogenes, whereas in humans, the gene family consists of around 400 functional and 600 pseudogenes (Gilad and Lancet 2003; Niimura 2009; Mainland et al., 2013; Hayden and Teeling 2014; Barnes et al., 2020). A given OSN expresses only one of them (Buck and Axel, 1991).

OSNs with the same receptor protein send their axon to the same location in the brain, namely one of more than 5,000 glomeruli in the human olfactory bulb, compared to about 2000 glomeruli in mice and rats (Maresh et al., 2008; McGann, 2017). The axon terminal of each OSN forms synaptic contacts with dendrites of olfactory bulb interneurons (juxtaglomerular cells) and output neurons, the mitral and tufted cells. Mitral and tufted cells send their axon to higher order brain centers in cortical and limbic structures for further processing (Shepherd et al., 2004; Pashkovski et al., 2020). The connections of OSN axons to specific olfactory bulb glomeruli and the projection of olfactory bulb neurons to other brain regions are key factors for odorant specific signal processing as a collaborative function of olfactory and limbic systems (Ennis et al., 2007; Pashkovski et al., 2020). OSN receptor proteins respond to odorants at the dendritic end of the cell and act as guidance molecule at the axonal end toward the olfactory bulb (Zamparo et al., 2019). OSNs are embedded in the pseudostratified ciliated, columnar olfactory neuroepithelium next to supporting and microvillar cells as well as basal stem cells. OSNs have a relatively short life span (1–2 months) because of their vulnerable, exposed location in the nasal cavity and are continuously replenished by younger OSNs generated from multipotent basal stem cells (Farbman, 1990; Calof et al., 1998; Cowan and Roskams, 2002; Schwob, 2002; Beites et al., 2005; Brozettei et al., 2020). The life-long addition and elimination of OSNs in the nasal olfactory epithelium allows for adaptive structural responses to sensory experience, learning, and recovery after injury (Sultan-Styne et al., 2009).

**FIGURE 1:**
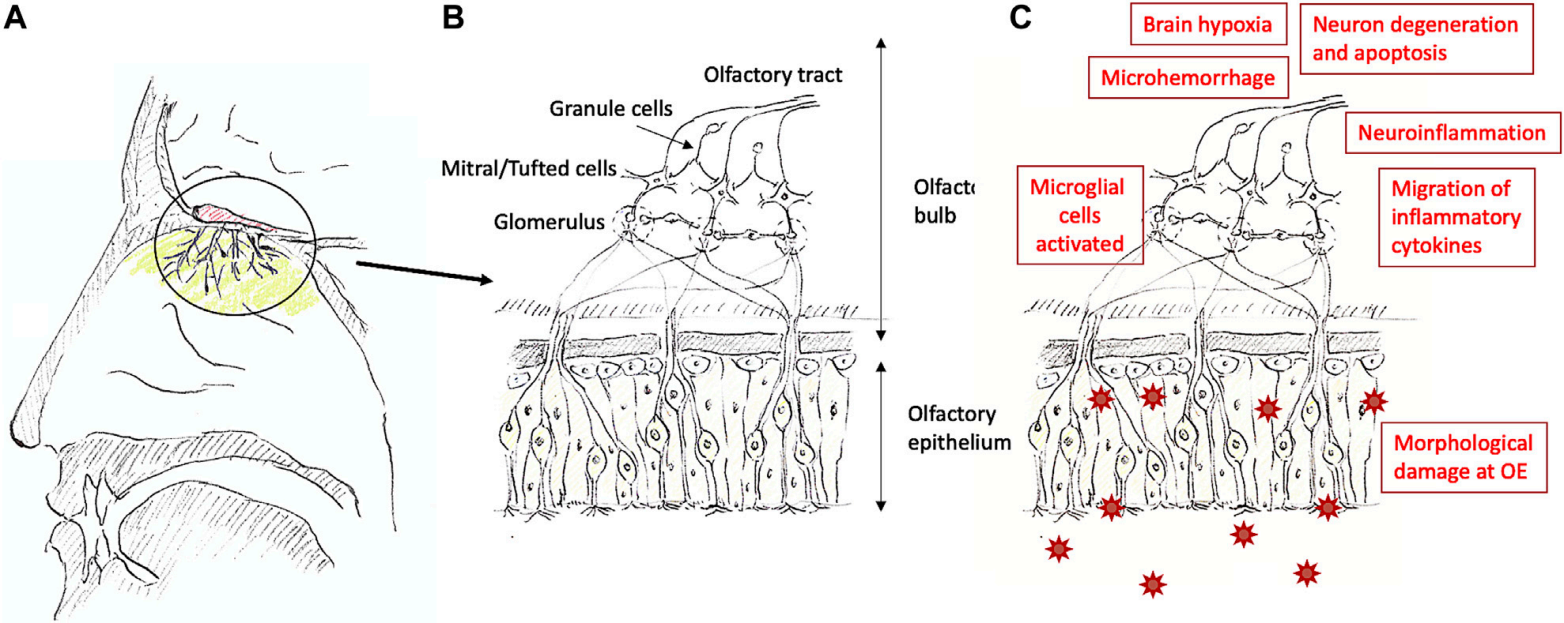
Nose **(A)**, a summary of the olfactory system **(B)**, and possible causations causing olfactory dysfunction **(C)**. The olfactory epithelium is located at the upper end in the nasal cavity [colored with light green in **(A)**], and the olfactory bulb is located above the area [colored in red in **(A)**]. The structure of the olfactory epithelium toward the olfactory bulb [the circled area is shown in **(A)**] is shown in **(B)**. At the olfactory epithelium, there are supporting cells [rather square-ish cells without axons extending to the olfactory bulb in **(B)**], Bowman’s glands [not drawn in **(B)**], and basal cells [round cells in **(B)**] other than the olfactory sensory neurons [cells with cilia and extending axons toward the olfactory bulb in **(B)**]. SARS-CoV-2 enters the supporting cells through ACE2 and cause morphological damage, inflammation, and further migrates to the brain (Meinhardt et al., 2020). Olfactory dysfunction can be caused by multiple factors as shown in **(C)**.

Several clinical manifestations have been described for olfaction. The most prominent ones are anosmia and hyposmia which have many etiologies, including trauma, chronic sinusitis, neoplasms, and respiratory viral infections such as rhinovirus and SARS-CoV-2 (Saltagi et al., 2021). Hyposmia manifests itself as a slightly diminished sense of smell, whereas anosmia refers to a complete loss of smell (Hummel et al., 2017). Research in response to the recent COVID-19 pandemic has revealed that a sudden loss of smell can be a symptom and strong predictor of SARS-CoV-2 infection and even occur in otherwise asymptomatic patients (Moien et al., 2020; Parma et al., 2020; Tong et al., 2020; Gerkin et al., 2021). Recent studies have provided hypotheses on the causation of anosmia, which includes neuroinflammation, inflammatory cytokines, neural degeneration and apoptosis, brain hypoxia, and morphological damage (de Melo et al., 2021; Xydakis et al., 2021; Rutkai et al., 2022) (Figure 1). The severity of the olfactory dysfunction and the time length it takes for the recovery could be determined by the types of factors involved and their severity.

## Olfactory Training and Its Procedure

A landmark study was published in 2009 (Hummel et al., 2009). In this study, the participants were patients with olfactory dysfunction (older than 18, average age 57.8, range 27–79, 33 women and 23 men). The participants sniffed four types of odorants in the morning and evening, about 10 s each time, for 12 weeks. The four types of odors were 1) phenyl ethyl alcohol (PEA), which represented the smell of rose, 2) eucalyptol, representing eucalyptus, 3) citronellal, representing lemon, and 4) eugenol, representing cloves. These four odorants were selected based on the odor prism, as mentioned above, although in this study the individual odorants and not the whole essential oils were used. Before the 12 weeks’ training period and after it, the olfactory sense was tested using Sniffin’ Sticks. The odor discrimination and odor identification, other than odor threshold, were measured. Although the age range was wide, including individuals of 79 years old, the age did not significantly affect the results. Odor thresholds were improved in response to PEA, citronellal, and eugenol but not to eucalyptol. Improvement of discrimination and identification of odors were not clear compared to the improvement of odor thresholds, which suggests that sensing the existence of a smell is a separate aspect from recognizing what the odors are, or it requires more training in order to identify what the odors are.

The concentration that will bring the best results is perhaps one of the key factors that needs to be determined. This was tested in a study comparing higher and lower concentrations (Damm et al., 2014), using the same four odorants as Hummel et al. (2009). The group tested with higher concentration used the odorants at neat concentrations, i.e., without dilution, whereas the group tested with lower concentrations used diluted odorant samples. In order to determine the dilution rate, the threshold concentration of the odorants in healthy women (*n* = 50, age 20–25 years) were first obtained, then the 10th percentiles of the threshold concentration for these odorants were calculated, and eventually a 0.0001% dilution of neat concentration was determined to be used for the lower concentrations (Damm et al., 2014). There were slight changes in the procedure from Hummel et al., 2009. The participants sniffed each of the four odors for 15 s (one cycle) and repeated this for one more cycle as one session. Every day, they carried out an olfactory training in the morning and in the evening, thus twice daily. This made the whole daily exposure to each of the 4 odorant to be 15 s twice, for a total of 30 s daily, thus, for the 4 types of essential oils totally 120 s of exposure per day. The duration of the olfactory training was also 18 weeks for one session compared to the 12 weeks in the study by Hummel et al. (2009), and it was repeated twice, totaling 36 weeks. The olfactory sense was tested three times, i.e., before training, after 18 weeks, and after 36 weeks. The olfactory sense significantly improved in the group that used higher odor concentrations, i.e., the neat concentration. The authors concluded that olfactory training is a safe method to improve olfactory function and that a higher odor concentration is recommended (Damm et al., 2014).

Another question that needs to be addressed is the location where olfactory training evokes changes, i.e., whether the improvement is a “relearning at the level of brain circuitry” or “regeneration of the peripheral olfactory system”, i.e., the olfactory epithelium or both. Although this can be different depending on the patient and/or the disease that caused the olfactory dysfunction, recent studies using functional magnetic resonance imaging (fMRI) have shown that the effects of olfactory training [using the same procedure and odorants as Hummel et al. (2009)] were especially strong in hyposmia patients compared to anosmia patients after at least 24 weeks of training (Pellegrino et al., 2019). The test stimuli used were the smell of coffee and peach. Participants were asked to verbally rate the intensity of the smell, pleasantness, and the identity of the smell. Rating of the intensity of smells significantly improved both in the hyposmia and anosmia patients, whereas there was no significant improvement in the pleasantness, and the results on discriminating the smells were not clear (Pellegrino et al., 2019). Interestingly, other than the right dorsal anterior cingulate, in the hyposmia patients, the brain regions that mostly showed improvements in odor-evoked activation were on the left side of the brain (the Broca’s area, the left angular gyrus, the left medial frontal gyrus, and the left superior frontal gyrus). The increase in the activation in the areas related to semantic cognition in the hyposmia patients suggests the effects of olfactory training on the improved cognitive processing by olfactory training. The experimental procedure that requests the participants to verbally report may have had some influences on activating these areas due to the necessities of verbally describing the smells as well. These areas involved in semantic cognition in the brain were not activated in the anosmia patients, indicating the difficulties in sensing the smells and suggesting the lack of semantic cognitive processing, although olfactory training had some positive effects on anosmia patients as well. The only part that showed more odor-evoked activation after olfactory training in the anosmia patients was the right superior frontal gyrus (Pellegrino et al., 2019). Previous studies have shown that the right superior frontal gyrus is involved in translating conflict anticipation to the control of impulsive response (Hu et al., 2016). It could be that the activation of this area is related to the anticipation in the anosmia patients to sense some smells and suppressing the impulsive responses because of the difficulties in sensing.

In another study using fMRI, the volume of grey matter in the brain related to the limbic system and the thalamus was found to be reduced in patients with olfactory dysfunction, and olfactory training significantly improved the volume at these regions (Gellrich et al., 2018). In their study, they examined the volume of the grey matter of normosmia controls (age 45–69; 17 females and 14 males) and that of the patients with hyposmia (age 38–80, 16 females and 14 males). Patients were examined before and after olfactory training. The experimental group, i.e., hyposmic patients, went through olfactory training for 12 weeks twice a day, whereas the control group did not undergo olfactory training. The patients’ olfactory test scores significantly improved after the olfactory training, and the volume of grey matter in the hippocampus, thalamus, and cerebellum showed increases, but there were no differences between the groups (patients vs. control) in the volume of the olfactory bulb.

In a study of patients with Parkinson’s Disease, which is known to cause olfactory dysfunction, the same methods as Hummel et al. (2009) were used (Haehner et al., 2013). The patients who went through olfactory training showed significantly higher overall “Threshold-Discrimination-Identification (TDI)” scores. An interesting difference from Hummel et al. (2009), in which the participants did not have Parkinson disease, was that the scores on discrimination showed the strongest difference after olfactory training, whereas the scores on threshold and identification were not different from the control group (Haehner et al., 2013).

Since the publication of the landmark paper on olfactory training (Hummel et al., 2009), various protocols have been used to study olfactory training. A longer time period of training was found to produce larger improvement (Konstantinidis et al., 2016), and switching stimulus odors had better results (Altundag et al., 2015). Using a higher concentration of odors showed better improvements than using a lower concentration of odors (Damm et al., 2014). Comparative studies have shown that patients who went through olfactory training had 2.77 higher odds of improving the olfactory sense compared to the control (Kattar et al., 2021), especially on olfactory discrimination and olfactory identification (Pekala et al., 2016).

It is important to determine how this improvement is taking place. One hypothesis is that it helps in rewiring the neural network in the brain. However, it is also possible that the chemical constituents of the essential oils used in olfactory training facilitate the regeneration of olfactory neurons, and thus facilitate the recovery of the olfactory sense. We are not aware of any study that summarizes the chemical constituents of the essential oils used in olfactory training, specifically focusing on the bioactive properties of these chemical constituents. In the following section, we will summarize the chemical constituents of clove, eucalyptus, lemon, and rose, and discuss the possibilities that the bioactive properties of the chemical constituents of these essential oils have some roles in facilitating the recovery of the impaired olfactory sense.

## Chemical Constituents of the Essential Oils Used in Olfactory Training

The chemical constituents of essential oils vary by geographical location, weather, part of the plants the extract was made, and by the methods used in making the extracts (Koyama and Heinbockel 2020). This indicates that the major constituents of essential oils need to be always determined and confirmed in order to be aware of any differences and changes that can affect their efficacy. In the tables below we show these regional differences in the same essential oil extracts.

### Clove

Eugenol is one of the major chemical constituents of clove. Yet, the percentage of eugenol in comparison to other major chemical constituents in clove varies from 55.6% (Amelia et al., 2017) to as high as 88.59% (Chaieb et al., 2007) (Table 1). In the same country of Indonesia, the percentage of eugenol was 55.6% at Java compared to 74.64% at Manodo. Eugenyl acetate was also 8.7% at Manodo, which was less than half of the percentage of 20.54% in the clove oil from Java, Indonesia (Amelia et al., 2017). In addition to the large differences in the percentage of the same chemical constituent included in the oils, there are large differences in the major chemical constituents as well. In the study by Amelia et al. (2017), eugenol, eugenyl acetate, and caryophyllene were the 3 top major constituents in clove essential oils from Java and Manodo, Indonesia. Caryophyllene was found at trace levels as in other reports (Amelia et al., 2017).

**TABLE 1:** Major chemical constituents of clove plants and essential oils.

Data source	Source of samples	Major chemical constituents
Amelia et al. (2017)	From clove bud	Clove from Java, Indonesia: eugenol 55.60%, eugenyl acetate 20.54%, caryophyllene 14.84%, *α*-humelene 2.75%, *β*-elemene 0.04%, *α*-cadinene 0.05%, ledol 0.06%; from Manodo, Indonesia: eugenol 74.64%, caryophylene 12.79%, eugenyl acetate 8.70%, *α*-humelene 1.53%, *β*-gurjunene 0.04%, *γ*-cadinene 0.03%, humelene oxide 0.05%; comparison with other papers, eugenol, 47.60–89.20%, caryophyllene, trace level to 35.40%, eugenyl acetate, 1.20–20.54%, *α*-humulene, trace level to 2.75%
Chaieb et al. (2007)	From clove flower bud essential oil	Eugenol 88.59%, eugenyl acetate 5.62%, *β*-caryophyllene 1.39%, 2-heptanone 0.93%, ethyl hexanoate 0.66%, *α*-humulene 0.20%, calacorene 0.11%, calamenene 0.11%
Jirowetz et al. (2006)	From clove leaf essential oil	Eugenol 76.8%, *β*-caryophyllene 17.4%, *α*-humulene 2.1%, eugenyl acetate 1.2%, caryophyllene oxide 0.4%, methylchavicol 0.2%
Uddin et al. (2017)	From clove buds oil in Bangladesh	m-eugenol 69.44%, eugenyl acetate 10.79%, caryophyllene 6.8%, tyranton 7.78%, trace amounts of other constituents <1%

Although the percentages fluctuate, it is possible to state that the major chemical constituents of clove are eugenol, eugenyl acetate, and, depending on the location, caryophyllene. Eugenol (Ma et al., 2018) and eugenyl acetate are known to have anti-inflammatory effects (Saraphanchotiwitthaya et al., 2019). In *in vitro* studies, eugenol and eugenyl acetate suppressed IFN*γ*, IL-2, IL-10 levels (Saraphanchotiwitthaya et al., 2019), and in *in vivo* studies, eugenol suppressed TNF*α*, IL-1*β*, IL-6, and NF-kBp65 expression (Ma et al., 2018). Among other chemical constituents, *α*-humelene is found commonly as a chemical constituent, although the percentage is low (Jirowetz et al., 2006; Chaieb et al., 2007; Amelia et al., 2017; Uddin et al., 2017). *α*-Humulene is also known to have anti-inflammatory effects, suppressing the IL-5, CCL11, leukotriene B4 levels, and NF-kB and AP-1 activation (Sa et al., 2015; Nuutinen, 2018; Kim et al., 2020). These studies suggest that utilization of clove essential oil can have the effect of suppressing inflammation, although there could be differences in the extent of the effect depending on the percentages of the chemical constituents with anti-inflammatory effects in the oil.

### Eucalyptus

The chemical constituents of eucalyptus oil vary extremely largely, depending on the species of *Eucaylptus*, the part of the plant used, and the region they were harvested (Table 2). There are about 300 species of *Eucalyptus* plants and about 20 of them are used in extracting oils (Barbosa et al., 2016). 1,8-Cineole (also called eucalyptol) and *α*-pinene are major constituents of eucalyptus plants although percentages vary depending on the species and other factors. 1,8-Cineole and *α*-pinene are well known for their anti-inflammatory effects. Both 1,8-cineole and *α*-pinene significantly suppressed the level of pro-inflammatory cytokines, for example, IL-1*β*, IL-6, and TNF*α* (Caceres et al., 2017; Salehi et al., 2019a). In addition, *p*-cymene, limonene, spathulenol, *α*-terpineol, and borneol were found in eucalyptus but at lower percentages than 1,8-cineole and *α*-pinene (Dogan et al., 2017). Spathulenol and limonene were detected in leaves but not in fruits (Dogan et al., 2017). These chemical compounds suppress inflammation and the expression of pro-inflammatory cytokines (Sa et al., 2015; do Nascimento et al., 2018; Kim et al., 2020). These studies suggest that eucalyptus contains multiple anti-inflammatory chemical compounds, although the extent of their anti-inflammatory effects can be different because the percentage varies depending on the *Eucalyptus* species and the part of the plant used to generate the extract.

**TABLE 2:** Major chemical constituents of eucalyptus plants and essential oils.

Data source	Source of samples	Major chemical constituents
Barbosa et al. (2016)	Review paper	Major chemical constituents in the extracts from leaves are terpenes and terpenoids at various concentrations. The concentrations vary among the species of *Eucalyptus* and the location they were harvested. 1,8-Cineole most often is the most major chemical constituent in *E. camaldulensis*, *E. cinerea*, and *E. globulus* (percentages vary from as low as almost 10% to as high as over 90.0%), whereas citronellal is the major chemical constituent in *E. citriodora* (percentages varied from as low as almost 20% to as high as over 90%). *α*-Pinene is the major chemical constituent in *E. saligna* (from 24.4 to 45.1%), depending on the location the plants are harvested
Dogan et al. (2017)	Leaf Fruit	1,8-cineole 14.1%, p-cymene 42.1%, *α*-pinene 12.7%, *α*-terpineol 10.7%, limonene 5.5%, borneol L 5.5%, spathulenol 3.2% 1,8-cineole 34.5%, p-cymene 30.0%, *α*-terpineol 15.1%, *α*-pinene 9.0%, borneol L 5.3%, *γ*-terpinene 5.1%, spathulenol none, limonene none
Salehi et al. (2019b)	Review paper	Percentages are not shown
Sebei et al. (2015)	Review paper	Comparing the percentages among species, 1,8-cineole (49.07–83.50%) and *α*-pinene (1.27–26.35%) were the two major chemical constituents

### Lemon

Limonene is the chemical constituent of lemon essential oils with the highest percentage which varies from 31.5 to 69.9% (Table 3). *β*-Pinene is the second most prominent chemical constituent as indicated in a study by Pucci et al. (2020) with a percentage of 10.88%. It was among the major constituents in the essential oil from lemon leaf in a study by Klimek-Szczykutowicz et al. (2020). Lemon contains many chemical compounds with anti-inflammatory effects: *β*-caryophyllene, geraniol, limonene, linalool, myrcene, *α*-pinene, *β*-pinene, (E)- *β*-ocimene, sabinene, *γ*-terpinene, and terpinen-4-ol (Koyama et al., 2021 for review). The high percentage of limonene suggests that the anti-inflammatory effects could be mediated mainly by limonene, but the large number of chemical constituents with anti-inflammatory effects in lemon also suggests the possibility of synergetic effects by different chemical compounds through the activations of separate receptors and channels. For example, *β*-caryophyllene is known as a ligand of cannabinoid receptor 2 (Gertsch et al., 2008), limonene is known to activate TRPA1 channels (Terada et al., 2019), geraniol suppresses the potassium ion channel KV1.3 (Ye et al., 2019), linalool activates TRPA1 and TRPM8 (Riera et al., 2009) and suppresses potassium ion channel KV1.3 (Ye et al., 2019). Geraniol has binding affinity to the receptor binding domains (RBD) of SARS-CoV-2 (Kulkarni et al., 2020). These studies indicate that lemon has a strong potential in facilitating recovery from olfactory dysfunction through its anti-inflammatory effects and because it contains geraniol as one of its chemical constituents.

**TABLE 3:** Major chemical constituents of lemon plants and essential oils.

Data source	Source of samples	Major chemical constituents
Klimek-Szczykutowicz et al. (2020)	Essential oil of pericarp Essential oil of leaf	Limonene 69.9%, p-menta-3,8-diene 18.0%, *β*-pinene 11.2%, *γ*-terpinene 8.21%, myrcene 4.4%, sabinene 3.9%, geranial 2.9%, neral 1.5%, linalool 1.41%, *α*-pinene 1.1%, *α*-thujene 1.1%, *β*-bisabolene 0.5%, (E)-*β*-ocimene 0.4%, geraniol 0.2%, *β*-caryophyllene 0.2% limonene 31.5%, sabinene 15.9%, citronellal 11.6%, linalool 4.6%, neral 4.5%, geranial 4.5%, (E)-*β*-ocimene 3.9%, myrcene 2.9%, citronellol 2.3%, *β*-caryophyllene 1.7%, terpinen-4-ol 1.4%
Luciardi et al. (2021)	Essential oils	Limonene 59.14%, *γ*-terpinene 10.48%, *β*-pinene 15.41%, sabinene 1.76%, *β*-myrcene 1.65%, *α*-pinene 1.64%
Pucci et al. (2020)	The whole fractions	Limonene 67.1%, *β*-pinene 10.88%, *γ*-terpinene 9.32%, *α*-pinene 1.81%, geranial 1.72%, sabinene 1.83%, myrcene 1.57%

### Rose

Phenyl ethyl alcohol (PEA) has been considered to be the major chemical constituent of rose water. However, studies have shown that PEA is the major chemical constituents in plants but it is actually not the major chemical constituent in rose essential oils (Verma et al., 2010) (Table 4). The major chemical constituents in rose essential oils are citronellol and geraniol (Verma et al., 2010). These two chemical constituents of rose essential oils are responsible for their pharmacological activities such as anti-depressant, hypoglycemic, anti-inflammatory, analgesic, antioxidant, and antimicrobial effects (Akram et al., 2019). As stated above, geraniol has not only anti-inflammatory effects but also binding affinity with the RBD of the S-glycoprotein of SARS-CoV-2 (Kulkarni et al., 2020), which suggests that rose essential oil can have anti-inflammatory effects as well as anti-SARS-CoV-2 effects.

**TABLE 4:** Major chemical constituents of rose plants and essential oils.

Data source	Source of samples	Major chemical constituents
Akram et al. (2019)	Oil Absolute rose Hydrosol	Geraniols (5.5–18%), *β*-citronellol (14.5–47.5%), nonadecane (10.5–40.5%) heneicosane, ethanol (0–13.43%), geraniol (3.71%), citronellol (9.91%), nonadecane (4.35%), phenylethylalcohol (78.38%) nerol (16.12%), phenylethyl alcohol (23.74%), citronellol (29.44%), geraniol (30.74%)
Mileva et al. (2021)	Oils	There are large differences among geological differences and species differences; geraniol (17.60–30.98%), nerol (4.36–10.10%), citronellol (9.22–28.72%), n-nonadecane (8.10–22.67%), n-heneicosane (5.00–10.21%)
Ryu et al. (2020)	Oils	There are large differences among breeds (mutants) of roses
Verma et al. (2010)	From bud, half bloom, full bloomEssential oils	Phenyl ethyl alcohol (PEA) 66.2–80.7%, other major chemical constituent, although much less in the percentages, were citronellol (1.8–5.5%) and geraniol (4.4–7.9%). Major chemical constituents were citronellol (15.9–35.3%), geraniol (8.3–30.2%), nerol (4.0–9.6%), nonadecane (4.5–16.0%), and heneicosane (2.6–7.9%), and not PEA (0.6–2.9%)

### Bioactive Properties of the Major Constituents of Clove, Eucalyptus, Lemon, and Rose

In the four essential oils typically used in the olfactory training, many chemical constituents with anti-inflammatory effects are present (Table 5). However, there are very few chemical constituents with binding affinity to SARS-CoV-2 (only geraniol has been identified so far, Table 5). In the rose essential oil, the percentage of geraniol is high, which suggests that rose can be an adequate choice for olfactory training of COVID-19 patients with olfactory dysfunction. The lack of chemical compounds with binding affinity to SARS-CoV-2 in other essential oils suggests that these other oils may be effective in facilitating recovery from anosmia/hyposmia, if there are no or only few remaining SARS-CoV-2 viruses and if inflammation is the main reason causing the olfactory dysfunction. Recent studies have shown that in the post-COVID-19 patients with long-term anosmia/hyposmia, the proinflammatory cytokines were upregulated in the olfactory mucosa (de Melo et al., 2021). This suggests that inflammation could be the key in causing the COVID-19 induced anosmia/hyposmia, and utilization of essential oils with anti-inflammatory chemicals constituents could help in facilitating recovery from anosmia/hyposmia. However, the same study also showed that, although RT-PCR tests on the nasopharyngeal samples were negative, these patients all had detectable SARS-CoV-2 RNA in the olfactory mucosa samples (de Melo et al., 2021), suggesting that the remaining virus could be the reason of the long-term post-COVID-19 anosmia/hyposmia due to the inflammation that the virus is generating in the olfactory mucosa. For that reason, combinations of essential oils that contain chemical constituents with binding affinity to SARS-CoV-2 and others with anti-inflammatory effects might be most effective in facilitating recovery from COVID-19-induced anosmia/hyposmia. Therefore, we propose that it is important to test other combinations of essential oils, especially the combinations of essential oils with chemical constituents with anti-inflammatory effects and with binding affinity to SARS-CoV-2. For example, bitter orange, cinnamon, cypress, elderberry, geranium, lavender, lemongrass, licorice, mint, oregano, paper mulberry, peppermint, summer savory, tea tree, tea plant, thyme, and turmeric contain chemical constituents with binding affinity to SARS-CoV-2 as well as these with anti-inflammatory effects (Koyama et al., 2021). There are two major targets that the binding takes place. One target is the 3CL^pro^ or PL^pro^ proteases, which are involved in the replication of the virus, and the other is the RBD of the S-protein. The phytochemical compounds that possess binding affinity with the proteases are for example, curcumin, cyclocurcumin, (-)-epicatechin, epigallocatechin-3-gallate, kazinol J. The phytochemical compounds that possess binding affinity with the RBD of S-protein are, for example, anethole, carvacrol, cinnamaldehyde (E)-cinnamyl acetate, 1-menthol, 4-terpineol, thymol (Koyama et al., 2021). These differences the target region where binding takes place can generate differences in the effects. The binding to the proteases may suppress the replication, and thus they may facilitate recovery from COVID-19 and COVID-19 induced olfactory dysfunction if there are remaining virus and causing the dysfunction. The binding to the RBD region of the S-protein of the virus would hinder binding of the virus to ACE2, and thus suppress the entry of the virus into cells through ACE2. As there are many phytochemicals with anti-inflammatory effects, the utilization of the essential oils made from these plants has a possibility to facilitate the recovery from the olfactory dysfunction caused by COVID-19 by their effects on both inflammation and the virus. Importantly, as mentioned above, considering that there are large differences in the chemical constituent profiles in the same plants from multiple reasons, the determination of the chemical constituent profiles of the essential oils used in olfactory training and adjusting the concentrations to control the conditions would be necessary for the reliability in the effects.

**TABLE 5:** Chemicals compounds in the four essential oils with anti-inflammatory effects and binding affinity with SARS-CoV-2.

Chemical constituents with anti-inflammatory effect	Effects other than anti-inflammation	References
Borneol	Anti-viral (HSV-1 virus)	Armaka et al. (1999)
*β*-caryophyllene	Facilitate regeneration, enhance cell proliferation/migration; analgesic	Koyama et al. (2019)
1,8-cineole (eucalyptol)	—	—
Citronellol	Activate peroxisome proliferator-activated receptor (PPAR) alpha and gamma	Katsukawa et al. (2011)
p-cymene	—	—
Eugenol	—	—
Eugenyl acetate	—	—
Geraniol	Anti-angiogenic, anti-cell proliferative, apoptosis-inducing effects Vinothkumar et al., (2012); anti-ulcerogenic effects de Carvalho et al. (2014); Binding affinity to SARS-CoV-2 RBD of S-glycoprotein Kulkarni et al. (2021); activate peroxisome proliferator-activated receptor (PPAR) alpha and gamma Katsukawa et al. (2011)	Vinothkumar et al. (2012), de Carvalho et al. (2014), Kulkarni et al. (2021), Katsukawa et al. (2011)
*α*-humulene	—	—
Limonene	Anti-tumorigenesis	—
Linalool	Analgesic	—
Myrcene	Analgesic	—
(E)-*β*-ocimene	—	—
*α*-pheliandrene	—	—
α-pinene	—	—
Sabinene	—	—
Spathulenol	Dnti-nociceptive effects	Dos Santos et al. (2020)
*γ*-terpinene	—	—
